# Force Identification Based on Response Signals Captured with High-Speed Three-Dimensional Digital Image Correlation

**DOI:** 10.3390/s23020799

**Published:** 2023-01-10

**Authors:** Krzysztof Mendrok, Ángel J. Molina-Viedma, Elias López-Alba, Francisco A. Díaz Garrido, Lukasz Pieczonka

**Affiliations:** 1 Department of Robotics and Mechatronics, AGH University of Science and Technology, Al. A. Mickiewicza 30, 30-059 Krakow, Poland; 2 Departamento de Ingeniería Mecánica y Minera, Campus Las Lagunillas, Universidad de Jaén, 23071 Jaén, Spain

**Keywords:** dynamic testing, force identification, digital image correlation (DIC), modal filter, structural dynamics

## Abstract

Structural Health Monitoring (SHM) systems allow three types of diagnostic tasks to be performed, namely damage identification, loads monitoring, and damage prognosis. Only if all three tasks are correctly fulfilled can the useful remaining life of a structure be estimated credibly. This paper deals with the second task and aimed to extend state-of-the-art in load identification, by demonstrating that it is feasible to achieve it through the analysis of response signals captured with high-speed three-dimensional Digital Image Correlation (HS 3D-DIC). The efficacy of the proposed procedure is demonstrated experimentally on a frame structure under broadband vibration excitation. Full-field vibration displacement signals are captured with the use of two high-speed cameras and processed with 3D-DIC. Loads are identified with two different algorithms based on inverting the Frequency Response Function (FRF) matrix and modal filtration (MF). The paper discusses both methods providing their theoretical background and experimental performance.

## 1. Introduction

Knowledge of the forces acting on a structure is critical at both the design and operational stages. At the design stage, knowledge of the design loads to which the designed structure is to be subjected allows for optimizing the geometry in order to ensure safety without oversizing. During operation, knowledge of the real loads acting on a structure allows for the detection of shocks and overloads, which can be used to assess structural health and predict the useful remaining life of a structure. This can significantly reduce the risk of failure and extend the time between planned repairs. Knowledge of load history, therefore, significantly increases safety and reduces the operating costs of technical infrastructure. The loads acting on a structure can be measured directly, e.g., with piezoelectric force sensors, or indirectly, based on the strain values measured with strain gauges or fiber optic sensors. It is also possible to identify loads by measuring the response in the form of acceleration, velocity, or displacement of vibrations. This load identification method is usually cheaper and easier to implement, although, obviously, more computationally complex.

Force identification methods can be classified according to the domain in which they operate, and the nature of the algorithm used [1]. The first classification distinguishes two categories: time domain and frequency domain identification methods. The second classification distinguishes three groups: deterministic, statistical, and artificial intelligence-based methods. Within these three groups, there are many effective force identification algorithms, an overview of which can be found in [1,2,3].

In many identification methods, numerical conditioning improves with an increased number of implemented response signals [4]. This further results in an improvement in the accuracy of the identified force spectrum. Therefore, measuring responses in a large number of points significantly improves the efficacy of load monitoring. On the other hand, the necessity of measuring the responses in many points significantly increases the cost of the measurement system, its degree of complexity, and its susceptibility to failure.

Full-field vision measurements provide a viable solution to this problem by allowing the acquisition of responses with high spatial resolution, while retaining the measurement system’s simplicity. Additionally, the vision measurements are noncontact, and due to this fact, they do not introduce any structural changes to the test object. An application of vision measurements for force identification in the time domain is discussed in [5]. In this work, the authors employed a marker tracking algorithm on a simply supported beam to register the motion of 30 discrete points. Further enhancement could be obtained with the use of Digital Image Correlation (DIC), which provides real full-field displacement measurement offering much higher spatial resolution and accuracy when compared to marker tracking algorithms [6]. Recently, DIC has been gaining popularity in applications related to dynamic measurements of vibrations and modal analysis. This is thanks to modern high-speed cameras, which allow for high frame rate acquisition with high spatial resolutions. The capabilities of high-speed DIC have been recently assessed in different works. Many of them exploit its high spatial resolution to determine operational deflection shapes at specific frequencies. It has been employed to study nonlinear deformations [7], for highly curved geometries using multi-view systems [8,9], for full-field strain prediction [10] or to reveal subtle motion in combination with phase-based motion magnification [11,12]. High-speed DIC has also been successfully employed for full modal identification, including natural frequencies and damping ratios [13,14,15]. Hence, new applications have arisen from these methodologies with different purposes, like Finite Element (FE) model updating [16,17], Structural Health Monitoring (SHM) of wind turbine blades [18] or exploiting the dense measurement grid for damage location using modal filtration [19] and the mode shapes curvature [20].

This paper presents a novel approach to the identification of loading force spectra through the analysis of response signals captured with high-speed three-dimensional Digital Image Correlation (HS 3D-DIC). Two different force identification methods are compared, namely, methods based on the Frequency Response Function (FRF) matrix inversion and modal filtration (MF). Experimental validation of the proposed approach was performed on a laboratory-scale frame structure forced by an electrodynamic shaker. Response signals were acquired with two high-speed cameras.

## 2. Materials and Methods

### 2.1. Overview of Force Identification Methods

To keep the focus on the main topic of this work, the review of the existing force identification methods is limited to deterministic algorithms operating in the frequency domain. In many practical cases, including wear monitoring or transfer path analysis, precise knowledge of the excitation force time history is not necessary. Additionally, in machines where loads have a harmonic character (e.g., are generated by an imbalance of rotating parts), the frequency spectrum carries more information. The frequency domain methods are usually simpler to apply and need less computational power. These advantages are significant when it is necessary to perform force identification in real time.

### 2.2. Inversion of the FRF Matrix Method

The method is one of the most frequently used in practice and, therefore, the most widely presented in the literature [21,22,23]. This is due to its simplicity and effectiveness in terms of accuracy of identification. In order to identify the excitation force vector *F*(*ω*), it is necessary to measure the response of the system *Y*(*ω*) in the number of n points. This number should be greater than the number of identified forces. Then, assuming the FRF matrix *H*(*ω*) is known, the following equation is used:

(1) $$F\left(\omega \right)={\left[H\left(\omega \right)\right]}^{+}\cdot Y\left(\omega \right)$$

**Figure 1:**
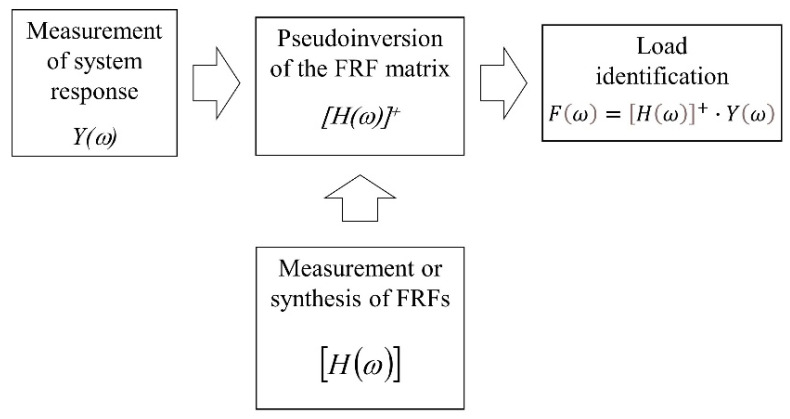
Block diagram of the FRF matrix inversion method.

The FRF matrix [*H*(*ω*)] is not square with a size of (*n × m*), and it cannot simply be inverted. Therefore, a pseudo-inversion algorithm should be used in the form of Singular Value Decomposition (SVD) or the Moore–Penrose method. Sometimes the FRF matrix is unavailable. In such a case, it can be synthesized based on an FE model [3] or a modal model [22] of the object. The block diagram of the method is shown in Figure 1.

### 2.3. Modal Filtration Method

Another method is based on modal filtration, which allows decomposing of the response of the system into components related to subsequent modes [24]. Modal filtration, i.e., the transition from physical to modal coordinates, is carried out according to the formula:

(2) $$\left\{\eta_{r}\left(\omega \right)\right\}={\left\{\psi_{r}\right\}}^{T}\cdot {\left\{Y\left(\omega \right)\right\}}_{}$$

where: *η_r_* is *r*-th modal coordinate, *ψ_r_* is the *r*th reciprocal modal vector (vector orthogonal to all modal vectors except the *r*-th one).

The application of modal filtering to force identification consists of four steps [25]:

Modal filtration of the system response vector *Y*(*ω*), i.e., the transition from physical to modal coordinates *η*(*ω*).
Determination of the number of uncorrelated excitation forces.
Location of these unknown forces.
Determination of the force spectra from the matrix equation:
  (3) $$\left[F\left(\omega \right)\right]={\left[\Phi \right]}^{T}{}^{+}\cdot \left[\Pi \left(\omega \right)\right]$$
  where: [Φ] is the modal vectors matrix, [Π] is the matrix that contains vectors of weighted modal coordinates *η*(*ω*).

**Figure 2:**
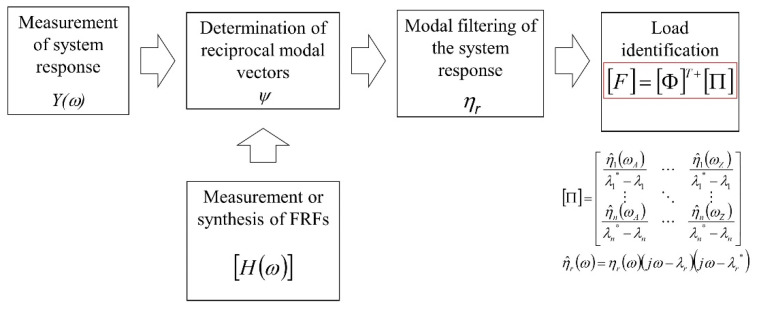
Block diagram of the modal filtration method.

The block diagram of the method is shown in Figure 2.

Modal filtration can also be performed based entirely on operational response data [26,27].

### 2.4. Dynamic Stiffness Method

The dynamic stiffness characteristics can also be applied for force identification [28]. It is applicable when the interface element between the exciter and the structure has lower stiffness than the rest of the system. For such an element, the dynamic stiffness characteristic *K*(*ω*) is measured. In order to estimate the excitation force with this method, displacements of both sides of the connecting element have to be measured i.e., on the exciter side—*Ys*(*ω*) and on the structure side—*Yt*(*ω*):

(4) $$F\left(\omega \right)=K\left(\omega \right)\cdot \left(Y_{s}\left(\omega \right)-Y_{t}\left(\omega \right)\right)$$

The measurement of both displacement signals *Y_s_*(*ω*) and *Y_t_*(*ω*) must be done simultaneously.

### 2.5. Method Based on the Mutual Energy Theorem

This method of excitation force identification is based on the Heaviside mutual energy theorem [29]. The algorithm is also used in acoustics to identify the sources of sounds [30]. A series of preliminary experiments is required in which the forces are successively applied to the system output measurement points. Responses to these excitations are measured at the location where the force acts. The monitored force is derived from the following formula:

(5) $$F_{a}={\left[\dot{Y}\right]}^{-1}F{\dot{Y}}_{a}$$

where *F_a_* is the identified force vector, $\left[\dot{Y}\right]$ are the matrix of response velocities measured in consecutive experiments and consecutive points, $F{\dot{Y}}_{a}$ is the vector of power calculated as a product of known forces from consecutive experiments and velocity caused by an unknown identified force.

For the identification of the loading force spectrum based on vision-based measurement data, we decided to use deterministic methods with well-proven performance. The methods based on the inversion of the FRF matrix and on the modal filtration were selected as the methods of choice for further analysis. The other two methods had to be discarded as they did not comply with the experimental arrangements. The method based on the dynamic stiffness was rejected because the shaker was attached to the test structure with a stiff, rather than compliant, stinger, and, hence, the basic assumption of this method was not fulfilled. The method based on the mutual energy theorem requires many experiments, which was considered impractical for engineering applications, and the method was also excluded from further analysis.

### 2.6. Digital Image Correlation (DIC) Background

DIC is a full-field optical technique used for displacement measurements in structural or mechanical components on their surfaces [31]. It works by analyzing a sequence of images of the specimen from a reference state (undeformed) to a final state (deformed) and tracking the displacement of points from one image to the other. A region of interest is defined in the reference image and divided into evenly spaced squares called subsets. The process is performed by matching the light intensity in the subset area corresponding to the initial and final position of each subset. To evaluate the degree of similarity between the reference and the deformed subset, a correlation criterion is employed, and the subset is identified in the final image as that which maximizes the correlation coefficient. DIC also considers that the original square reference subset might have a distorted shape due to the deformation of the specimen. For this purpose, a shape function is assigned to the subset, which relates the local pixel coordinates in the reference subset to the coordinates in the image after deformation. With suitable calibration, the displacement vector is computed through the coordinates of each subset in the reference and deformed states, creating a dense map. When a sequence of images is analyzed, every image corresponding to a state of deformation is correlated to the reference image in this way.

For a successful matching, every subset must be unique; hence the specimen surface must have a random gray intensity distribution (a random speckle pattern), which deforms together with the specimen. The speckle pattern can be the natural texture of the specimen surface or artificially made, usually by spraying a random black speckle over a white background applied on the specimen surface.

**Figure 3:**
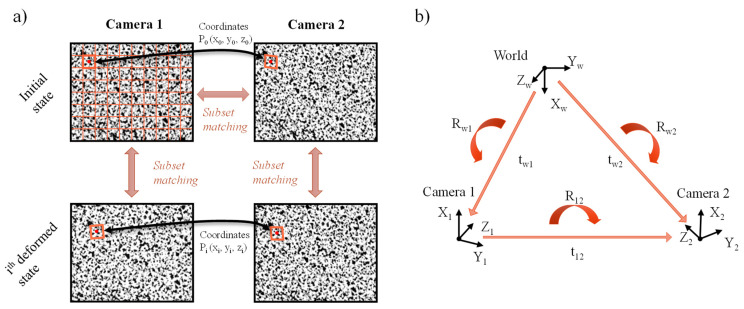
(**a**) Image correlation procedure for subset matching in stereoscopic measurements. (**b**) Translational and rotational transformations of the coordinate systems employed for 3D-DIC.

The 3D-DIC version employs stereovision and triangulation to retrieve 3D displacements from the image sequences of two cameras. For this purpose, an additional correlation is required in the first step to match the subsets of the reference image of both cameras, as shown in Figure 3a. Triangulation is then performed to determine the 3D coordinates of each subset according to the following parameters of the stereo system obtained in the calibration procedure: intrinsic (related to the lens and the camera) and extrinsic (transformations between the camera coordinate systems and the world system) parameters, as illustrated in Figure 3b).

### 2.7. Experimental Arrangements

**Figure 4:**
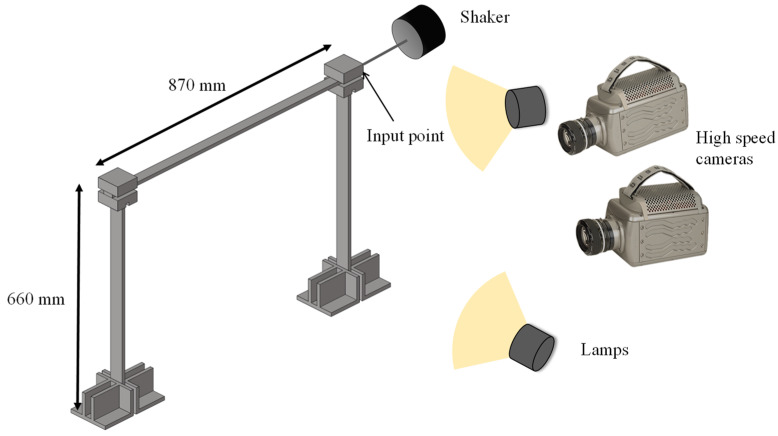
Scheme for broadband excitation of the frame structure and the optical arrangement for high-speed 3D displacement measurement.

The structure under test was a frame-like structure consisting of two vertical steel beams with a rectangular cross-section of 10 × 40 mm, and one horizontal aluminum beam with a rectangular cross-section of 10 × 30 mm. They were rigidly attached by two joint blocks, as shown in Figure 4. The frame was also rigidly fixed to the base. The structure was excited by an electrodynamic shaker with a stinger attached to the joint block on the right-hand side, through a force sensor registering the excitation force. The shaker was supplied with a white noise signal with a lowpass filter set to 256 Hz.

The whole structure was monitored by the vision system, including the joint blocks, in order to register the response at the input point. The main motion induced in the structure occurred in the horizontal plane; thus, the optical system was placed in front of this plane. The monitored surfaces were at different depth planes from this point-of-view, so the adequate estimation of the structure displacements had to be performed using a stereoscopic system for 3D-DIC, as illustrated in Figure 4. Two high-speed cameras, model *Phantom v9.1*, were employed, with a sensor size of 1632 × 1200 pixels. According to the excitation bandwidth, the frame rate of the cameras was set to 512 fps to fulfill the Nyquist–Shannon criterion. The exposure time was 400 ms, which was ten times shorter than the period of the highest frequency excited. Hence, blurring, due to the fastest movements, was avoided. To compensate for the short exposure time of the sensor, two light sources were employed, which improved the quality and contrast in the images needed for the correlation procedure. The length of a recording sequence was limited to 3160 images due to the internal storage of the high-speed cameras. Therefore, two consecutive sequences were recorded to provide data for normal mode identification and force identification, respectively. Using the abovementioned test setup parameters, the acquisition from the high-speed cameras and the force sensor were synchronized to ensure a proper estimation of the modal response of the structure to a particular input.

**Figure 5:**
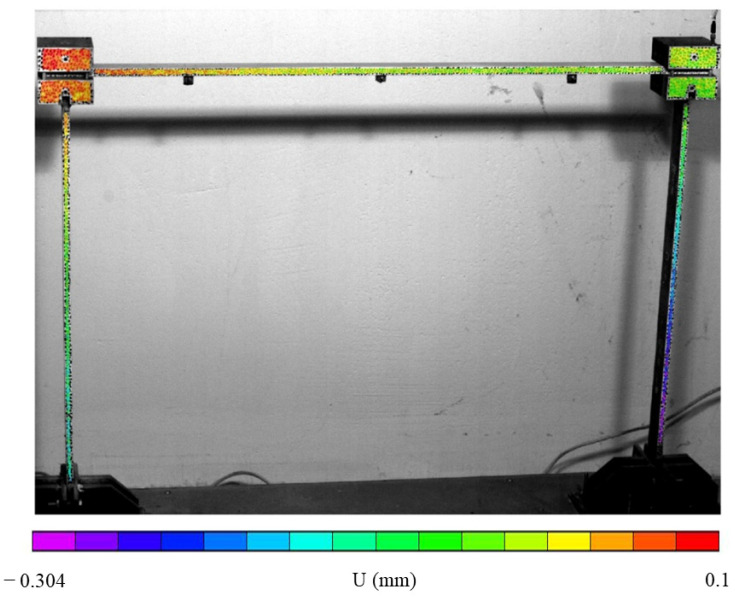
Horizontal displacement maps measured on the frame structure surface at a certain instant under broadband excitation.

As a metallic structure, its surface was reflective and had no adequate random pattern for DIC. Hence, the observed surfaces were coated with matt white paint background and black dots, producing a random speckled pattern. The DIC procedure was performed using 9 pixels subsets, in the regions of interest, with a 2 pixels step between them to increase the number of points in the narrow parts. The resulting measurement map for a particular time instant during the test can be observed in Figure 5.

For the prediction of input forces, the employed dataset consisted of only the measurement points of the right vertical beam and its corresponding joint block, where the excitation was applied. Under this excitation, the vertical beam experienced a typical 1D bending deflection, with negligible vertical displacement, whereas the block could be considered a rigid body, due to its high stiffness and location away from the fixed end of the vertical beam. Therefore, it could be assumed that there was no variation of the transverse deflection in thickness and the 2D maps of the horizontal displacements of the beam and the block were reduced to a 1D line of 493 virtual sensors, by calculating the mean value of the displacements in thickness (horizontal direction).

## 3. Research Methodology

As described in the previous section, two measurement sequences of 3160 samples each were acquired. They contained the reference excitation force signal, measured with the piezoelectric force sensor, and the vibration signal, measured with the high-speed vision system. The data were recorded in the time domain, but as explained in Section 2, the topic of the work was the application of identification algorithms operating in the frequency domain. Therefore, the waveforms were transformed to the frequency domain using the Fourier transform. The Fast Fourier Transform (FFT) [32] algorithm was implemented in the Matlab software. Based on the first sequences of data, frequency spectra of excitation forces, responses in the form of vibration displacements, and frequency response functions were estimated. The processing parameters were as follows: the H1 estimation method for FRFs, the number of averages—5, overlapping—50%, frequency resolution—0.5 Hz, frequency band—256 Hz. With the use of the abovementioned data, the modal model of the object was then identified. The bandwidth resulted directly from the sampling frequency of the measured signals (512 fps), the frequency resolution and overlapping were set so as to maximize the number of averaging for the lengths of recorded time histories. The modal model was necessary for the modal filter method but may also be helpful for the synthesis of unmeasured FRFs [22]. In Table 1, the modal model parameters obtained with the use of the PolyMAX algorithm [33] are presented.

**Figure 6:**
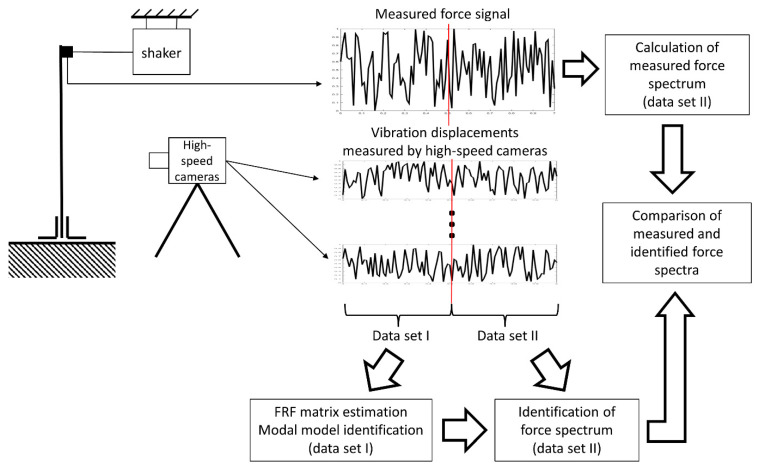
Experimental validation methodology.

The excitation force spectrum was identified in the next step, based on the responses from the second data sequence, and compared with the measured force spectrum. The procedure is shown in Figure 6. As a measure of the quality of identification, the value of Pearson’s correlation coefficient between both spectra and the average value of signal spectrum magnitude were assumed.

## 4. Results of Force Identification

### 4.1. Identification with Use of FRF Inversion Method

According to the procedure described in the previous section, after the estimation of the corresponding frequency characteristics, the FRF matrix (*H* in accordance with Equation (1)), with dimensions 493 × 1 (493 response signals × 1 identified excitation), and the response vector (*Y*), with dimensions 1 × 493, were compiled. It was decided to use all available response signals to minimize estimation errors, as shown in [4]. Then, for each frequency in the band, the spectral line of the excitation spectrum was calculated, according to Equation (1). For the sake of clarity, Equation (1) is shown below in expanded form:

(6) $$F\left(\omega_{n}\right)=\left[\begin{array}{c} H_{1,1}\left(\omega_{n}\right)\\ \vdots \\ H_{i,j}\left(\omega_{n}\right)\\ \vdots \\ H_{493,1}\left(\omega_{n}\right) \end{array}\right]\cdot \left\{\begin{array}{ccc} Y_{1}\left(\omega_{n}\right) & \ldots & Y_{493}\left(\omega_{n}\right) \end{array}\right\}$$

where: *H_i,j_*(*ω_n_*) is the *n*-th spectral line of the frequency response function between excitation in point *i* and response in point *j*.

**Figure 7:**
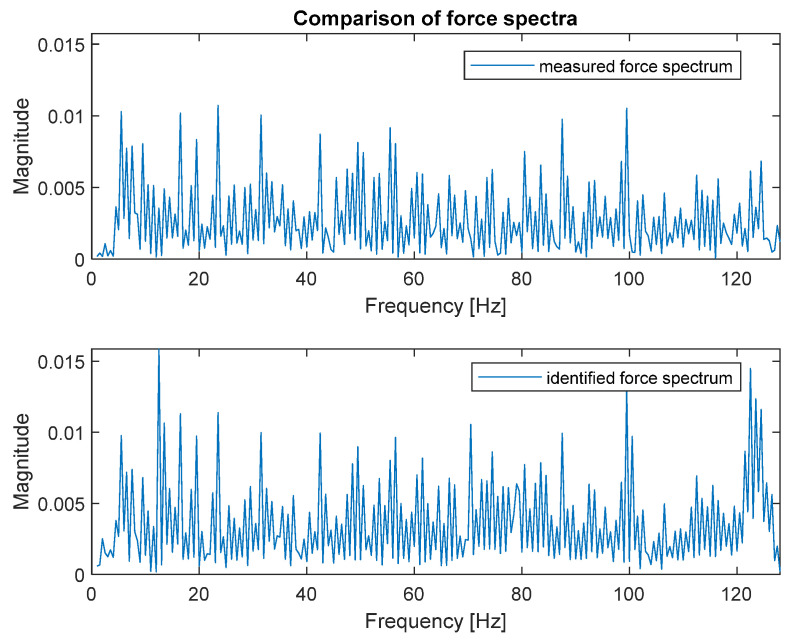
Comparison of measured (**top**) and identified (**bottom**) force spectra (FRF matrix inversion method).

The identified force spectrum, together with the reference spectrum obtained from the force sensor, are presented in Figure 7.

**Figure 8:**
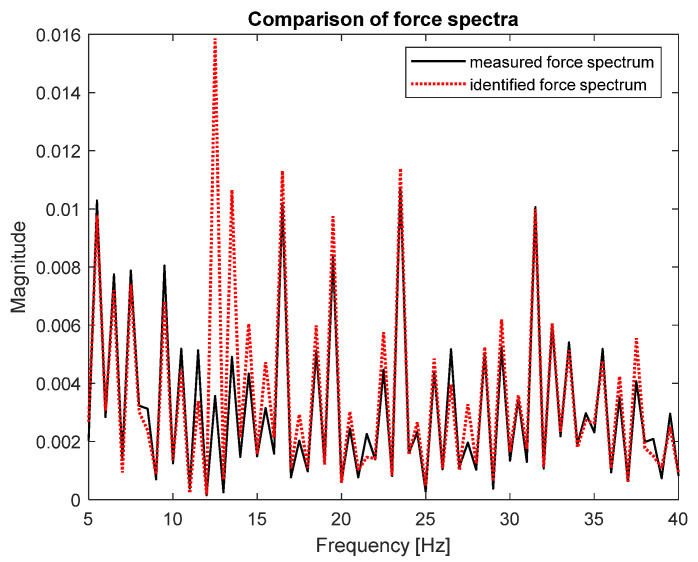
Comparison of measured (solid black line) and identified (dotted red line) force spectra—zoomed frequency range (FRF matrix inversion method).

As can be seen, both spectra are very similar; that is the reason they were placed on separate plots. Figure 8 presents both spectra in the narrower frequency range for detailed comparison.

The zoomed plot shows good quality of the identified force. The exception was the frequency of 12.5 Hz, where the first of the natural frequencies of the frame was located. There was a significant overestimation of the value of the identified force. This was in line with the analysis of errors from work [4]. It is worth noting that in the other natural frequencies, the identification errors were no longer that large. As a quantitative measure of the identification quality, the value of Pearson’s correlation coefficient between both spectra and the average value of signal spectrum magnitude were used. These are typical indicators used to assess the quality of identification for such signals. The Pearson’s correlation coefficient assesses the similarity of the shape of the compared spectra, and their average value determines the compatibility of the power of both signals. The results for both criteria are presented in Table 2.

### 4.2. Identification with Use of Modal Filter Method

**Figure 9:**
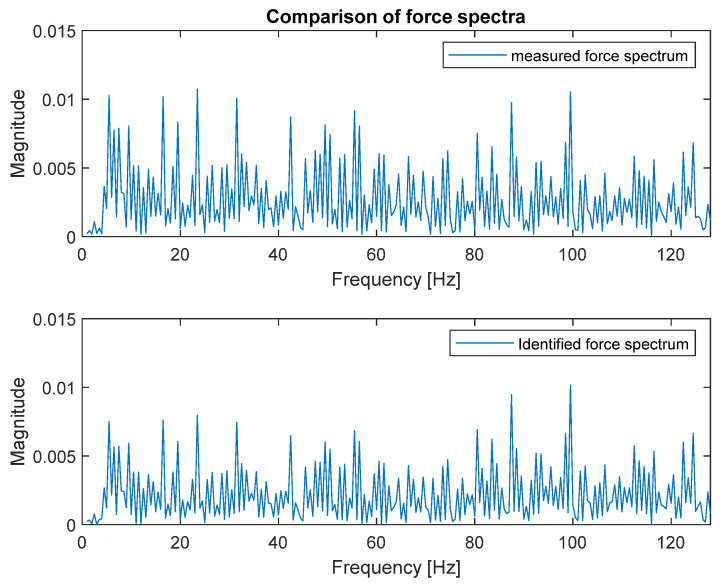
Comparison of measured and identified force spectra (modal filter method).

The modal model described in Section 3 (Table 1) was used to identify the modal filter. Six reciprocal modal vectors were estimated [24]. With their help, 493 spectra of output signals Y related to physical coordinates (measured with 3D-DIC) were transformed into six spectra of responses in modal coordinates. As in the previous section, all available response signals were used to improve the numerical conditioning of the problem and, thus, improve the accuracy of the estimated force. Then the Π matrix with dimensions of 6 × 511 (6 responses in modal coordinates × 511 frequency lines) was compiled, and the matrix F was calculated in accordance with Equation (3). Since there was only one excitation in a known location in the performed experiment, it was not necessary to determine the number of uncorrelated excitations or their locations. Thus, the column of matrix F related to the location of the excitement contained the spectral vector of that excitation. The obtained values of the magnitudes of the excitation spectrum were slightly underestimated. Therefore, an additional scaling factor was identified. For this purpose, the magnitude of the spectrum of the measured and identified force at five frequencies in the range was compared. The mean value of the obtained results amounted to 4.97, and was adopted as the scaling factor. The identified force spectrum, together with the reference spectrum obtained from the force sensor, are presented in Figure 9.

**Figure 10:**
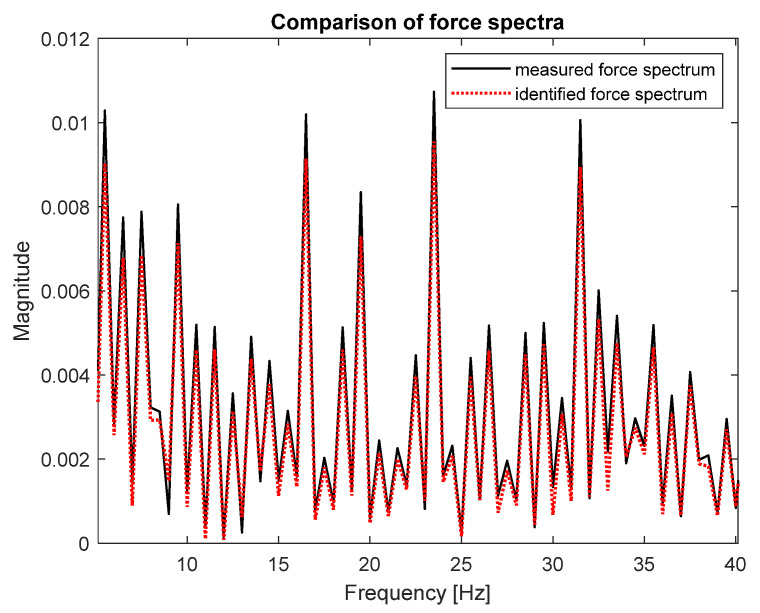
Comparison of measured and identified force spectra—zoomed frequency range (modal filter method).

In this case, the presented force spectra were even more similar than for the FRF-matrix-based inversion, and again, for that reason, they are presented on separate plots. Figure 10 presents both spectra in the narrower frequency range for detailed comparison.

## 5. Summary and Conclusions

Good quality of force spectrum identification could be seen for both methods, as shown in Figure 7 and Figure 9. The quantitative measure of the identification quality was obtained by calculating the value of Pearson’s correlation coefficient between both spectra and the average value of signal spectrum magnitude. The results for the FRF matrix inversion and the modal filter method are presented in Table 2.

The data presented in Table 2 confirm that it was possible to effectively identify the load on the basis of the response signals measured with the high-speed camera. Force identification methods operating in the frequency domain are often designed to estimate the order of magnitude of the load, so an accuracy of 70% was most acceptable.

Among the identification algorithms used, the one based on modal filtering worked better, although it required additional scaling, as discussed above. However, its superiority was confirmed by the value of the Pearson’s correlation coefficient and the percent relative error value. The worse performance of the method based on the FRF matrix inversion resulted from its large errors in the natural frequencies [4]. However, when analyzing the results in terms of the average value of the spectra, one should remember about the scaling factor used in the modal filtering method.

The paper demonstrated the application of vibration displacement measurements with high-speed 3D-DIC applied to identify the excitation force spectrum. Full-field vision measurements and two different input identification algorithms were evaluated and compared: FRF matrix inversion and modal filtration. In both cases, positive results were obtained, while greater accuracy of the force spectrum identification was obtained with the use of the modal filtration-based method. The use of vision measurements greatly facilitates and reduces the costs of the identification process. Based on the research, it can be seen that the monitoring of loads can now be carried out without the need to install a complex measurement system or interfere with the structure of the object.

## Figures and Tables

**Table 1:** Natural frequencies (NFs) and modal damping coefficients (MDCs) of the identified modal model.

Mode No.	NF [Hz]	MDC [%]
1	12.53	2.74
2	70.05	0.59
3	110.95	1.03
4	128.15	0.42
5	182.93	2.01
6	232.41	0.77

**Table 2:** Quantitative assessment of the considered force identification methods.

Identification Method	Compared Spectrum	Pearson’s Correlation Coefficient	Average Value of Spectrum Magnitude	Relative Error [%]
FRF matrix inversion	Measured force	0.80	0.0027	29.6
	Identified force		0.0035	
Modal filter	Measured force	0.96	0.0027	3.7
	Identified force		0.0026	

## Data Availability

Data is available on request.
